# Biocontrol Potential of an Endophytic *Streptomyces* sp. Strain MBCN152-1 against *Alternaria brassicicola* on Cabbage Plug Seedlings

**DOI:** 10.1264/jsme2.ME17014

**Published:** 2017-05-18

**Authors:** Naglaa Hassan, Satoko Nakasuji, Mohsen Mohamed Elsharkawy, Hushna Ara Naznin, Masaharu Kubota, Hammad Ketta, Masafumi Shimizu

**Affiliations:** 1 Laboratory of Plant Pathology, The United Graduate School of Agricultural Science, Gifu University1–1 Yanagido, Gifu 501–1193Japan; 2 Laboratory of Crop Production and Ecology, Graduate School of Bioresources, Mie University1577 Kurimamachiya-cho Tsu city, Mie 514–8507Japan; 3 National Institute of Vegetable and Tea Science (NIVTS), National Agriculture and food Research Organization (NARO)3–1–1 Kannondai, Tsukuba 305–8666Japan; 4 Department of Plant Pathology, Faculty of Agriculture, South Valley UniversityQena 83523Egypt; 5 Department of Agricultural Botany, Faculty of Agriculture, Kafrelshikh University33516 KafrelshikhEgypt

**Keywords:** *Alternaria brassicicola*, endophytic *Streptomyces*, *Brassica oleracea* var. *capitata*, plug seedling

## Abstract

In the present study, 77 strains of endophytic actinomycetes isolated from cabbage were screened in order to assess their biocontrol potential against *Alternaria brassicicola* on cabbage seedlings. In the first and second screening trials, cabbage seedlings pretreated with mycelial suspensions of each isolate were spray-inoculated with *A. brassicicola*. Strain MBCN152-1, which exhibited the best protection in screening trials and had no adverse effects on seedling growth, was selected for the greenhouse trial. In the greenhouse trial, cabbage seedlings, which had been grown in plug trays filled with soil mix containing spores of MBCN152-1 (1×10^8^ spores g^−1^ of soil mix), were spray-inoculated with *A. brassicicola* and grown in greenhouse conditions. MBCN152-1 reduced disease incidence and significantly increased the number of viable seedlings. The efficacy of MBCN152-1 against damping-off caused by seed-borne *A. brassicicola* was then evaluated. Cabbage seeds, artificially infested with *A. brassicicola*, were sown in soil mix containing MBCN152-1 spores. The disease was completely suppressed when infested seeds were sown in a soil mix blended with MBCN152-1 at 1.5×10^7^ spores g^−1^ of soil mix. These results strongly suggest that MBCN152-1 has the potential to control *A. brassicicola* on cabbage plug seedlings. MBCN152-1 was identified as a *Streptomyces humidus*-related species based on 16S rDNA sequencing. Scanning electron microscopy showed that the hyphae of MBCN152-1 multiplied on the surface of the seedlings and penetrated their epidermal cells. In conclusion, strain MBCN152-1 is a promising biocontrol agent against *A. brassicicola* on cabbage plug seedlings.

In modern agriculture, plug systems are widely used for the transplant production of vegetables and bedding plants worldwide (10, 11, 20). This production system provides numerous advantages to farmers: 1) plug seedlings are high quality and more uniform in size and condition, 2) these seedlings are adapted to mechanical transplantation, 3) root loss may be reduced during transplantation, and 4) the number of seedlings produced per unit of greenhouse space may be optimized (10). Given these major advantages, a number of crops are produced using this technique, particularly in Japan.

Cabbage (*Brassica oleracea* var. *capitata*) is one of the most popular leafy vegetables and plug systems are widely used for its seedling production (21, 39). Recent studies reported that many diseases affect cabbage plug seedlings in large commercial nurseries in Japan (17–19, 22). The incidence of damping-off, caused by *Alternaria brassicicola* (Schweinitz) Wiltshire, is the highest during the growing season (17, 18, 23, 25). *A. brassicicola* is a well-known seed-borne pathogen of *Brassica* plants worldwide (47, 52). Therefore, the primary inoculum of this disease on cabbage plug seedlings is also considered to be naturally infested seeds (21, 25). *A. brassicicola* initially forms dark gray to black necrotic lesions on the cotyledons and hypocotyls of the seedlings and finally causes damping-off. The conidia produced on the diseased seedlings rapidly disseminate to the neighboring healthy seedlings by the overhead irrigation system and possibly air currents. The transplantation of infected seedlings into fields may result in major economic losses for farmers because of decreased productivity and reduced quality. In Japan, the fungicide polyoxin is registered for use on cabbage plug seedlings. Polyoxin achieves excellent suppression; however, this fungicide is liable to commercial failure because of the emergence of resistant genotypes (13). Furthermore, the application of chemical fungicides in greenhouses may endanger the health of workers. Therefore, biological control using beneficial microbes is anticipated to provide an alternative or complementary strategy.

The abilities of a number of antagonistic or parasitic microbes such as *Trichoderma* spp., *Pythium* spp., *Pseudomonas* spp., and *Bacillus* spp. to control plant diseases have been investigated (1, 15, 16, 26). However, there have only been a few successes under field conditions due to several factors. One of the most crucial factors is considered to be the survival rate or population density of biocontrol agents on/in the host plants or soil following their application. When selected agents exhibiting strong biocontrol abilities *in vitro* are applied to fields, their population may often be decreased by various environmental and biological stresses such as desiccation, competence for nutrition, and interference by native microbes, which reduces their biocontrol effects.

Most actinomycetes are considered to have a saprophytic soil habitat. However, recent studies have shown that a number of actinomycetes inhabit plant tissues as endophytes (4, 44, 48, 51). Furthermore, some have attracted the attention of researchers as novel biocontrol agents (2, 7, 9, 30, 43). For example, Coombs *et al.* (7) reported that some endophytic actinomycetes exhibited significant biocontrol activities against *Gaeumannomyces graminis* and *Rhizoctonia solani* on wheat. Kunoh *et al.* showed that the tissue-cultured seedlings of ericaceous plants treated with endophytic *Streptomyces* spp. exhibited intense resistance against *Pestalotiopsis sydowiana* and *Phytophthora cinnamomi* (24, 30, 41). The capacity of these actinomycetes to inhabit host plant tissues will ensure their stable colonization and increase their potential biocontrol ability.

The aim of this study was to screen endophytic actinomycetes with a high potential for the biocontrol of *A. brassicicola* on cabbage plug seedlings.

## Materials and Methods

### Isolation of actinomycetes from cabbage plants

The actinomycetes examined in this study were isolated from surface-disinfected cabbage plants (*B. oleracea* var. *capitata*) harvested from experimental farms at Mie University (Takanoo-cho, Tsu-city) and the National Institute of Vegetable and Tea Science (Ano-cho, Tsu-city) in Mie Prefecture, Japan. At least 10 cabbage plants were sampled from each field and used for the isolation of actinomycetes. Isolation was conducted according to the method described in our previous study (40). Briefly, cabbage plants were washed with running tap water and dried at room temperature for 1 d. Small pieces of each organ (more than 50 pieces per plant) were surface disinfected with 1% sodium hypochlorite and 70% ethanol, followed by air drying in a laminar flow cabinet. These samples were grown on plates containing IMA-2 agar medium (40) or 1.5% water agar, both of which were supplemented with antibiotics (amphotericin B: 0.05 mg mL^−1^, rifampin: 0.5 mg mL^−1^, viccillin: 0.5 mg mL^−1^, Heritage [Syngenta Japan, Tokyo, Japan]: 0.3 mg mL^−1^) to prevent contamination with undesirable fungi and bacteria.

Approximately 1 month after the incubation, the actinomycetes that emerged from the samples were isolated and purified using the membrane filter method (35). Actinomycetes isolated from the samples were transferred onto autoclaved membrane filters (mixed cellulose ester, pore size=0.2 μm; Advantec, Tokyo, Japan) that were placed on the surface of IMA-2 agar medium. After an incubation at 30°C for 7–14d, the actinomycetes that penetrated into the medium through the membrane pores were transferred to mannitol/soya agar (MSA) medium (12) and cultured at 30°C until sporulation. The spores of each actinomycete were suspended in an appropriate amount of sterile distilled water (SDW) and spread onto the surface of Bennett’s medium (27), followed by an incubation at 30°C. Single colonies were then individually transferred to fresh MSA and incubated at 30°C until sporulation. Spores were harvested in 10% glycerin solution containing 10% dimethyl sulfoxide solution and then maintained at –80°C until used.

### Preparation of actinomycetes and fungal pathogen inocula

In the present study, mycelial and spore suspensions of actinomycetes were used as inocula in the experiments. Each mycelial suspension was prepared as follows: 100 μL of the stock solution of each actinomycete strain was added to 100 mL of IMA-2 liquid medium and incubated on a rotary shaker (200 rpm) at 30°C for 24 h. The number of colony-forming units per mL (CFU mL^−1^) in the mycelial suspensions (may include not only mycelia, but also spores) was counted using the dilution plating method. Spore suspensions were prepared in SDW using 2-week-old cultures grown on MSA at 30°C. Aerial mycelia and spores were scraped from the medium with sterile spatula and suspended in SDW. The suspension was then vigorously vortexed, sonicated for 30 s, and filtered through a funnel equipped with a folded sterile cotton cloth to remove mycelia. The spore concentration in the filtrate was assessed using a hemocytometer.

*A. brassicicola* was cultured on potato dextrose agar (PDA) at 25°C for 1 week. *A. brassicicola* conidia were suspended in an aqueous solution of 0.01% Silwet L-77 (Nippon Unicar, Kawasaki, Japan). The conidial concentration was measured using a hemocytometer and adjusted to 10^5^ or 10^6^ conidia mL^−1^, and the suspension was then used for inoculation.

### Screening trials

Seventy-seven actinomycete strains were used in the first screening trial. Seeds of cabbage (cv. Matsunami; Ishii Seed Growers, Shizuoka, Japan) were surface-disinfected by washing in running tap water for 10 min, before immersing in 1% sodium hypochlorite for 3 min and rinsing three times with SDW. Sterilized seeds were sown in 128-cell plug trays containing an autoclaved commercial soil mix (Napura, Yanmar, Osaka, Japan) and incubated in a controlled-environmental chamber (27°C, 12 h daylight). One d after sowing, a mycelial suspension (*ca.* 10^6^–10^7^ CFU mL^−1^) of each strain was dropped onto the seeds (1 mL per seed) and they were grown for a further 6 d. A challenge inoculation was performed by spraying a conidial suspension of *A. brassicicola* (10^5^ conidia mL^−1^) onto cabbage seedlings until run off occurred. The inoculated seedlings were incubated in a humid chamber (KCLP-1400 ICTS, NK system, Osaka, Japan) at >90% relative humidity and 27°C for 3 d, and then transferred to a controlled environmental chamber (27°C, 12 h daylight) for an additional 4 d. The degree of pathogen infection was assessed on seedlings at the end of the experiments and categorized into three levels: (i) healthy, (ii) slightly infected, and (iii) severely infected or dead. The percentage protection level was calculated using the following formula:

Protection percentage=(A-B)/(C-B)×100,

where A is the number of viable seedlings (including healthy+slightly infected seedlings) in the actinomycete treatment, B is the number in the control treatment, and C is the number of seeds sown. In the first screening trial, 12 seedlings were assayed using each strain.

Actinomycete strains with a protection level of >80% in the first screening trial were re-evaluated in the second screening trial. The experimental procedures employed in the second screening were the same as those in the first screening, except for the amount of the actinomycete inoculum applied and the number of seedlings used. In the second screening trial, each seed was treated with 3 mL of the actinomycete mycelial suspension and 96 plug seedlings were used for each strain. The protection level (%) was calculated as described above.

### Greenhouse trial

A spore suspension of strain MBCN152-1 was blended thoroughly with commercial soil (Napura, Yanmar) at a concentration of 1×10^8^ spores g^−1^ of soil mix. Cabbage seeds were sown into plug trays filled with MBCN152-1-blended soil and grown in a greenhouse at 24°C–37°C. Seven d after sowing, cabbage seedlings were spray-inoculated with a conidial suspension of *A. brassicicola* (10^5^ conidia mL^−1^) and placed in a humid chamber (KCLP-1400 ICTS, NK system) at >90% relative humidity and 27°C for 30 h, before growing in a greenhouse for 14 d. Seedlings grown in the untreated soil mix were also inoculated with *A. brassicicola* as a control treatment. At the end of the experiment, the percentage of viable seedlings (including healthy+slightly infected seedlings as a percentage of the number of seeds sown) and the percentage protection level were calculated as described above. Each treatment comprised three replicates and each replicate used 68 plug seedlings; thereby, making a total of 194 seedlings per treatment, and the experiment was repeated three times. Data were arcsine-transformed before the analysis; however, untransformed data are presented in the present study. Differences between the control treatment and MBCN152-1 treatment were analyzed using the Student’s *t*-test.

### Biocontrol efficacy of strain MBCN152-1 against seed-borne *A. brassicicola*

Cabbage seeds were artificially infested with *A. brassicicola* according to the following procedure. Seeds were surface-disinfected by immersing in 70% ethanol for 1 min and 1% sodium hypochlorite for 3 min, rinsed three times with SDW, and dried in a laminar flow cabinet for 30 min. Seeds were subsequently soaked in a conidial suspension of *A. brassicicola* (10^6^ conidia mL^−1^) for 2 h and then air-dried. An autoclaved commercial soil mix was blended thoroughly with the spore suspension of strain MBCN152-1 at different concentrations (1.5×10^5^, 1.5×10^6^, and 1.5×10^7^ spores g^−1^ of soil mix). The autoclaved soil mix was blended with SDW and used as a control treatment. Thirty infested seeds were sown into a Petri dish containing 20 g of soil, blended or not blended with strain MBCN152-1, and then grown in a controlled environment chamber (27°C, 12 h daylight) for 14 d. At the end of the experiment, the percentage of diseased seedlings was assessed. There were three replicates for each treatment and the experiment was repeated three times. Data were arcsine-transformed for the analysis; however, untransformed data are shown in the present study. An analysis of variance was applied, followed by separation of the means with Tukey’s HSD.

### *In vitro* antagonistic activity of strain MBCN152-1

The ability of strain MBCN152-1 to inhibit the growth of *A. brassicicola* was assessed using the dual culture technique. In this experiment, 1.5% water agar, IMA-2 agar, Bennett’s medium, and PDA were used for testing. A plug that was 0.8 cm in diameter and contained mycelia taken from a 1-week-old colony of the pathogen cultured on PDA was placed at the center of 9-cm plates of each agar medium and 5 μL of the strain MBCN152-1 spore suspension was placed at a distance of 3 cm from the fungus. The plates were incubated at 25°C for 10 d and fungal growth inhibition was monitored.

### Microscopic examination of conidial germination of *A. brassicicola* on cotyledon leaves

Cabbage seeds (cv. Matsunami) were surface-disinfected with ethanol and sodium hypochlorite as described above. Seeds were then sown in 128-cell plug trays containing an autoclaved soil mix (Napura) blended with a spore suspension of strain MBCN152-1 (1.5×10^7^ spores g^−1^ of soil mix) and incubated in a controlled-environmental chamber (25°C, 12 h daylight). Seven d after sowing, a 10-μL aliquot of the *A. brassicicola* conidial suspension (10^5^ conidia mL^−1^) was spotted on the surface of each cotyledon leaf, and then incubated in a moist transparent plastic box placed in the same controlled-environmental chamber. As a control, untreated seedlings were similarly inoculated with *A. brassicicola*. Six, 9, and 12 h after the inoculation, cotyledon leaves were detached from the seedlings and decolorized by placing them in acetic acid/100% ethanol (4:96 [v/v]) for 6 h, followed by a 15-min soak in 0.5% aniline blue in 0.1 M phosphate buffer (pH 8.5). The cotyledon leaves were then gently washed three times with 0.1 M phosphate buffer (pH 8.5), and the percentage of germinated conidia was assessed by microscopic observations of 30 conidia per cotyledon leaf. Six cotyledon leaves for each treatment were observed at each time point, and the experiment was repeated three times. Data were arcsine-transformed before the statistical analysis (Student’s *t*-test).

### Re-isolation of strain MBCN152-1 from cabbage seedlings

Ten seeds of cv. Matsunami were surface-disinfected as described above and sown in a translucent polycarbonate bottle (Culture bottle CB-3, AS ONE, Osaka, Japan) containing the autoclaved soil mix blended with the spore suspension of strain MBCN152-1 (1.5×10^7^ spores g^−1^ of soil mix). As a control, surface-disinfected seeds were sown in autoclaved soil mix without any treatment. Seven d after the incubation in a controlled-environmental chamber (25°C, 12 h daylight), the seedlings were uprooted and their cotyledon leaves were detached. Half of the cotyledon samples were then surface-disinfected with sodium hypochlorite and ethanol as described above and placed on 1.5% water agar in a Petri dish. The remaining half of the samples were placed on 1.5% water agar without surface disinfection. These plates were incubated at 30°C for 21 d. A total of 50 seedlings were used for each treatment.

### Scanning electron microscopy (SEM) of the colonization of cabbage seedlings by strain MBCN152-1

Seeds of cv. Matsunami were surface-disinfected as described above. Surface-disinfected seeds were coated with strain MBCN152-1 by rolling the seeds on a 2-week-old colony of the strain, and then sowing on 1% water agar in a translucent polycarbonate bottle (Culture bottle CB-3), followed by an incubation in a controlled-environment chamber (25°C, 12 h daylight). Seven and 14 d after sowing, seedlings were sampled from the bottles for SEM observations, which were conducted according to the method of Minamiyama *et al.* (31). Briefly, specimens were fixed with glutaraldehyde and osmium tetroxide, dehydrated with ethanol, freeze-fractured, critical-point dried, coated with gold, and observed by SEM.

### Identification of strain MBCN152-1

Strain MBCN152-1 was preliminarily identified based on taxonomic criteria, including its cultural and morphological characteristics on the International *Streptomyces* Project (ISP) No. 2–5 media recommended by Shirling and Gottlieb (45). The morphology of the strain grown on ISP-5 medium for 2 weeks was observed by SEM according to the method described by Shimizu *et al.* (43). The identity of the strain was confirmed by a 16S rRNA gene sequence analysis. The 16S rRNA gene was sequenced as described by Coombs and Franco (6). The sequence of the corresponding nucleotides was elucidated using an ABI PRISM 3100 Genetic Analyzer (Applied Biosystems, Foster City, CA, USA). The nucleotide sequence of the 16S rRNA gene for strain MBCN152-1 has been deposited in the DDBJ under accession no. LC034258. A homology search of the 16S rDNA sequence of strain MBCN152-1 was performed using the BLAST program via the DDBJ Web site (http://blast.ddbj.nig.ac.jp/blastn). A phylogenetic tree was constructed with CLUSTALW using the neighbor-joining method with Kimura’s two-parameter model (14).

## Results

### Isolation of actinomycetes from cabbage plants

More than 200 actinomycete colonies gradually emerged on the surfaces of the leaves and roots of surface-disinfected cabbage samples after *ca.* 1 month of incubation. In total, 178 strains were successfully isolated without contamination from other microbes. However, 101/178 strains exhibited poor mycelial growth and sporulation on agar media. Since these strains were not of practical use as biocontrol agents, they were excluded. Therefore, 77 strains with active growth were used in subsequent screening trials.

### Screening trials

**Fig. 1:**
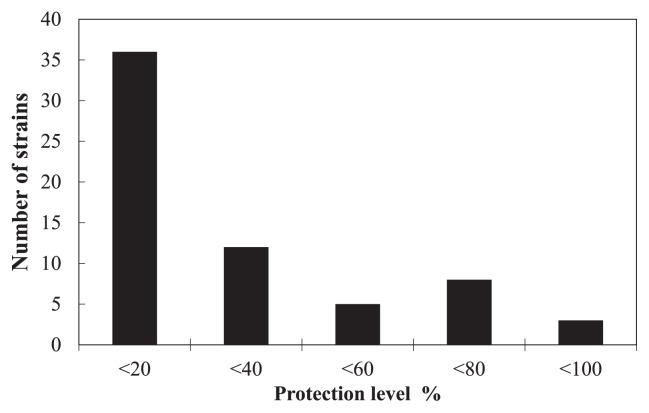
Number of actinomycete strains that achieved different levels of disease suppression (as percentages at 20% intervals) during the first screening trial.

In the first screening trial, we evaluated the control efficacy of 77 actinomycete strains against spray-inoculated *A. brassicicola*. Seven d after the challenge inoculation, most of the untreated seedlings exhibited severe symptoms or damping-off. In contrast, 11 strains suppressed the development of symptoms more strongly than the untreated control (*i.e.*, ≥60% protection level) (Fig. 1). Three strains (MBCY58-1, MBCN56-1, and MBCN152-1) exhibited strong control (*i.e.*, ≥80% protection level); thus, they were selected for the second screening trial.

**Fig. 2:**
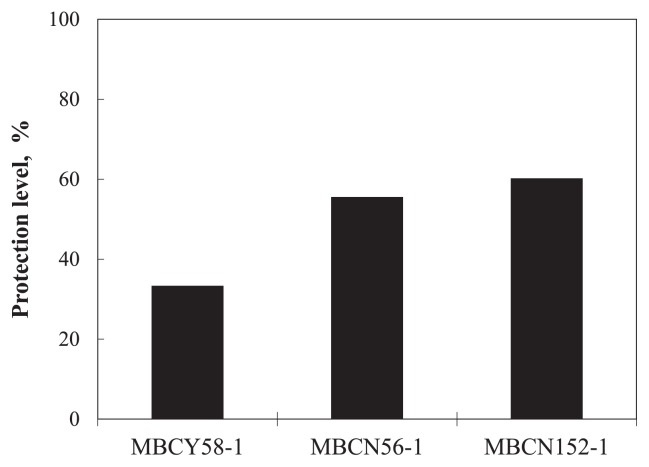
Effects of strains MBCY58-1, MBCN56-1, and MBCN152-1 on the percentage of viable seedlings in the second screening trial.

In the second screening trial, strains MBCY58-1, MBCN56-1, and MBCN152-1 achieved protection levels of *ca.* 33%, 56%, and 60%, respectively (Fig. 2). Although the protection level of strain MBCN56-1 was similar to that of strain MBCN152-1, the seed treatment with the former strain caused the abnormal growth of seedlings, including the slight shrinking of leaves and stunted growth (data not shown). Therefore, strain MBCN152-1 was selected as the final candidate for biocontrol.

### Greenhouse trial

**Fig. 3:**
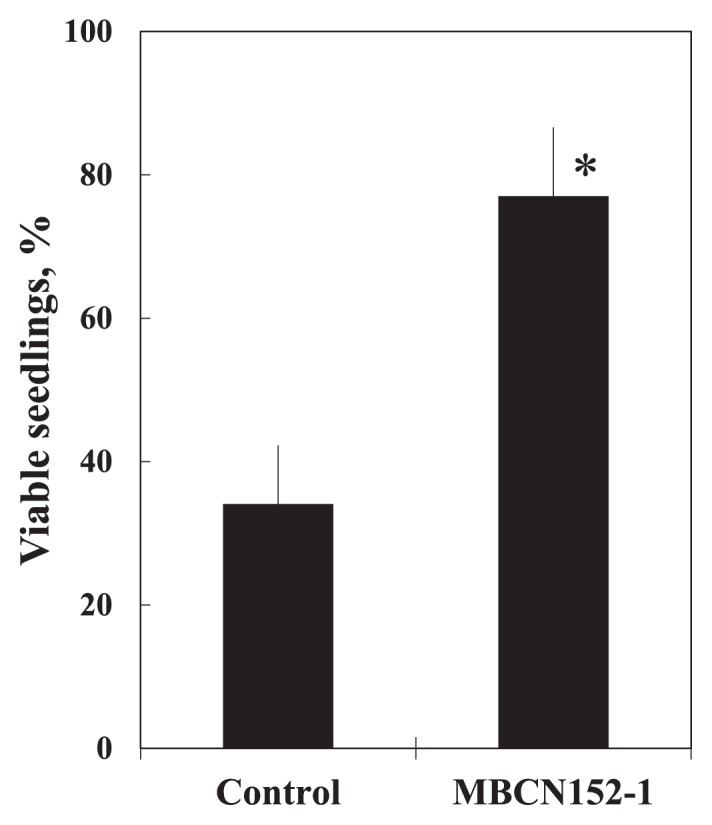
Effects of strain MBCN152-1 on the percentage of viable seedlings in the greenhouse test. Bars represent standard deviations. The asterisk indicates a significant difference from the control as calculated by the Student’s *t*-test (*P*<0.01).

The biocontrol effects of strain MBCN152-1 against spray-inoculated *A. brassicicola* was then assessed under greenhouse conditions. In the control, most seedlings were heavily diseased and only 34% remained viable 14 d after the pathogen inoculation (Fig. 3). In contrast, the treatment with strain MBCN152-1 suppressed the development of symptoms and a significant (*P*<0.01) increase was observed in the number of viable seedlings (viable seedlings=*ca.* 77%, protection level=*ca.* 65%).

### Effects of candidate strain MBCN152-1 on seed-borne *A. brassicicola*

**Fig. 4:**
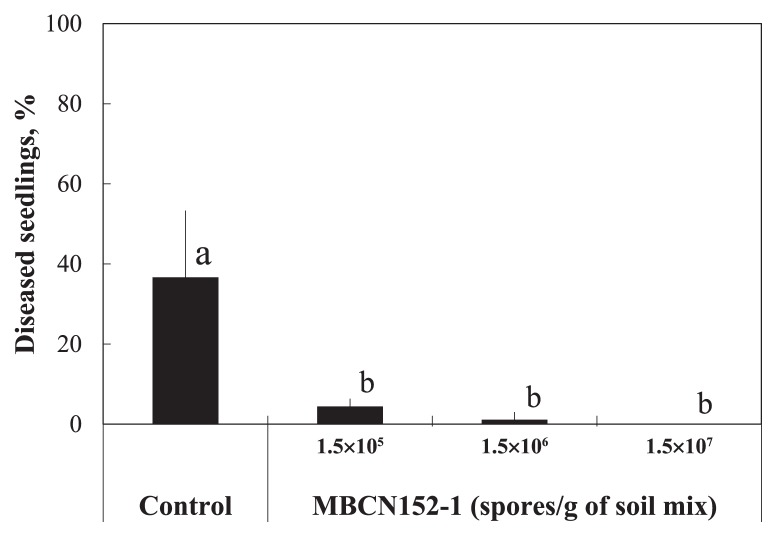
Effects of different concentrations of MBCN152-1 on cabbage seedlings after the artificial infestation of seeds with *A. brassicicola*. Bars represent standard deviations. Different letters indicate significant differences as calculated by Tukey’s HSD (*P*<0.05).

In order to evaluate the biocontrol effects of strain MBCN152-1 on seed-borne *A. brassicicola*, cabbage seeds were artificially infested with *A. brassicicola* and sown into soil blended with a spore suspension of strain MBCN152-1. The MBCN152-1 treatment reduced the incidence of seed-borne *A. brassicicola* disease (Fig. 4). In the control, *ca.* 37% of seedlings were infected by the pathogen and most exhibited damping-off. In contrast, the treatment with MBCN152-1 significantly (*P*<0.01) suppressed disease incidence, even at the lowest spore concentration (1.5×10^5^ spores g^−1^ of soil mix). The protection level increased with the spore concentration of strain MBCN152-1. The blending of 1.5×10^7^ spores g^−1^ in the soil mix reduced the number of diseased seedlings by 100% from the untreated control, followed by 1.5×10^6^ spores g^−1^ (*ca.* 98%) and 1.5×10^5^ spores g^−1^ (*ca.* 90%).

### *In vitro* antagonistic activity of strain MBCN152-1

Based on the dual culture results, strain MBCN152-1 did not inhibit the mycelial growth of *A. brassicicola* on the agar media tested (Table 1), thereby indicating that the strain does not produce any antibiotics that inhibit *A. brassicicola*.

### Microscopic examination of conidial germination of *A. brassicicola* on cotyledon leaves

**Fig. 5:**
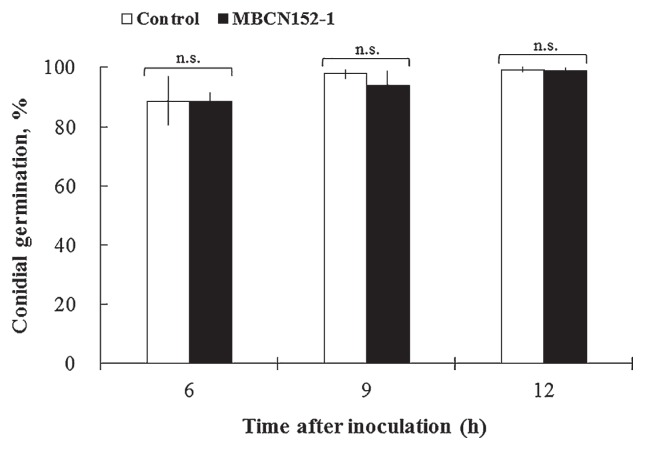
Percentage conidial germination of *A. brassicicola* on cotyledon leaves of MBCN152-1-treated cabbage seedlings and untreated seedlings. Bars represent standard deviations. Statistical analyses were performed with the Student’s *t*-test, n.s., not significant.

On the cotyledon leaves of untreated seedlings, 99.3% of *A. brassicicola* conidia germinated within 12 h of the inoculation (Fig. 5). Similarly, the percentage of conidial germination in the MBCN152-1 treatment reached 98.7% 12 h after the inoculation, indicating that the strain does not affect conidial germination on the surface of seedlings.

### Re-isolation of strain MBCN152-1 from cabbage seedlings

In order to analyze the ability to colonize cabbage seedlings, strain MBCN152-1 was re-isolated from either unsterilized or surface-disinfected cotyledon leaves of the seedlings grown in the soil mix containing the spores of strain MBCN152-1 (Table 2). The results obtained showed that the strain was re-isolated from all unsterilized cotyledon leaves on the 7th d after sowing. On the other hand, when surface-disinfected cotyledon leaves were incubated on water agar, the strain was re-isolated from 33% of samples, implying that some of its mycelia colonize endophytically within 7 d. In the untreated control, none of the actinomycetes colonies appeared from the samples regardless of surface disinfection.

### SEM of the colonization of cabbage seedlings by strain MBCN152-1

**Fig. 6:**
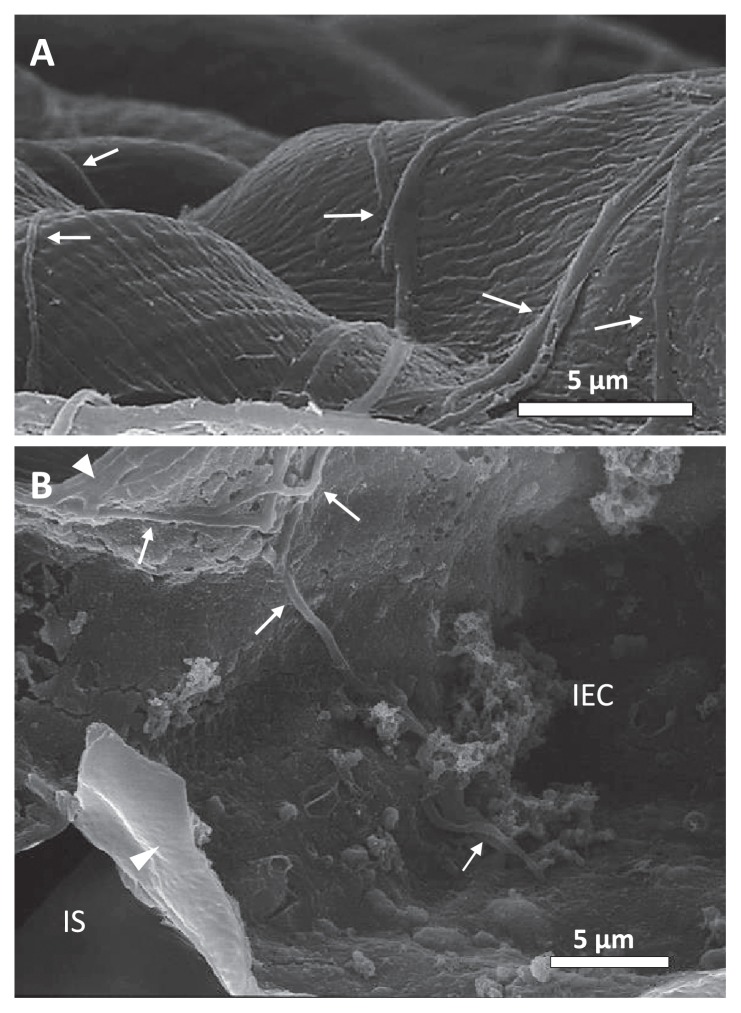
Scanning electron micrographs showing the outside and inside of cotyledon leaves from cabbage seedlings, which were grown from seeds treated with strain MBCN152-1. (A) Hyphae of strain MBCN152-1 growing on the leaf surface 7 d after sowing. (B) Hyphae of strain MBCN152-1 penetrating the cell wall (arrowhead) and growing inside an epidermal cell 14 d after sowing. Bars represent 5 μm. Arrows: hyphae of strain MBCN152-1. Arrowheads: cell walls of epidermal cells, IEC: inside epidermal cell, IS: intercellular space.

As shown in Fig. 6A, substrate mycelia extended over the surfaces of the seedlings pretreated with strain MBCN152-1. However, no mycelial extensions were observed on the untreated seedling surface (data not shown), which strongly suggests that they were strain MBCN152-1. Furthermore, observations of freeze-fractured samples showed that some mycelia of strain MBCN152-1 penetrated the cuticle layers and expanded into the underlying epidermal cells (Fig. 6B). These results clearly indicate that strain MBCN152-1 is an endophytic actinomycete.

### Identification of strain MBCN152-1

The culture characteristics of strain MBCN152-1 are summarized in Table 3. Strain MBCN152-1 grew well on each ISP media to form flat and powdery colonies. The aerial mycelia were grayish in color. The substrate mycelium was brownish or yellowish in color.

**Fig. 7:**
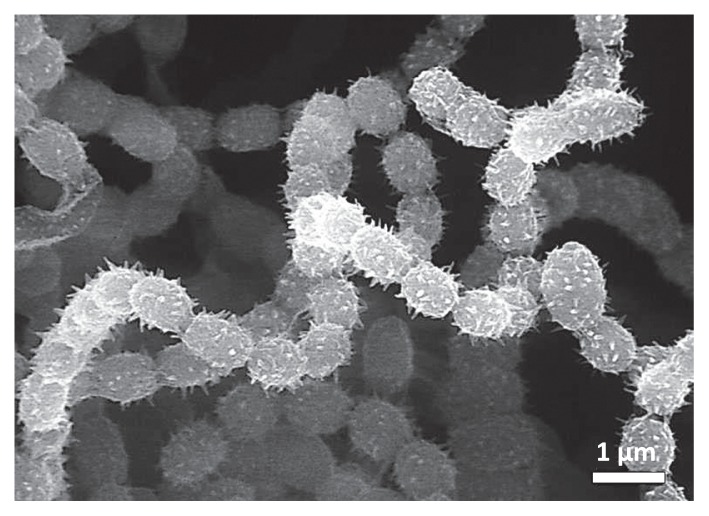
Scanning electron micrograph of spore chains in strain MBCN152-1. The bar represents 1 μm.

SEM showed that the spore chains of strain MBCN152-1 that formed on ISP-5 medium were spiral with >20 spores per chain (Fig. 7).

**Fig. 8:**
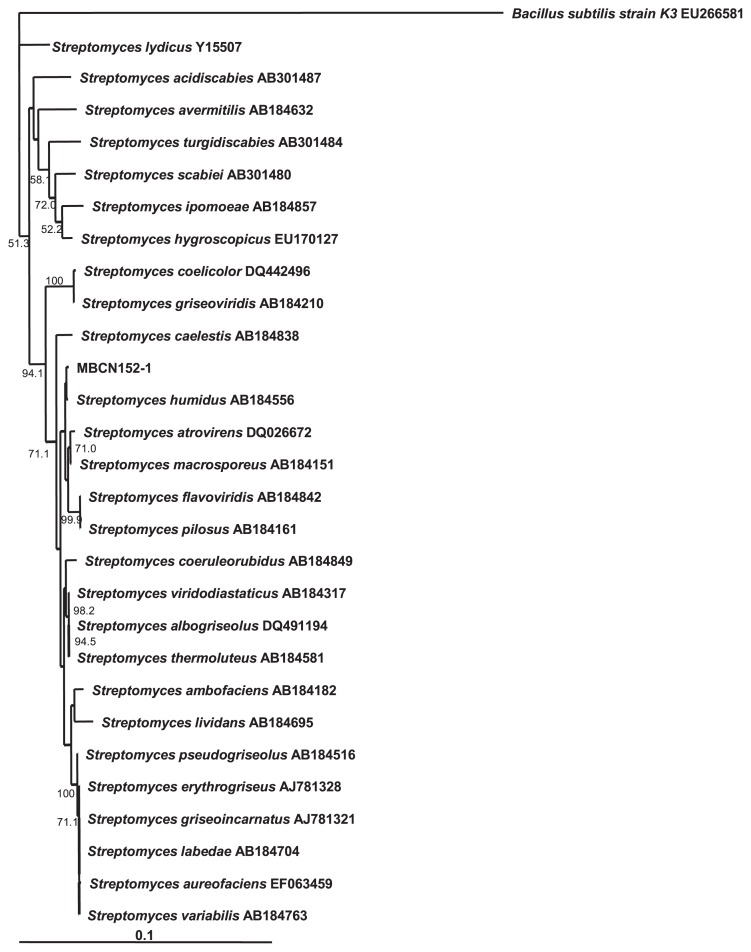
Neighbor-joining tree based on 16S rRNA gene sequences showing the position of strain MBCN152-1 versus related organisms. The numbers at the nodes indicate the levels of bootstrap support (%) based on 1,000 reassembled datasets, in which only the branches with >50% bootstrap support are labeled. The scale bar at the bottom indicates genetic distance units based on Nei’s genetic distance.

The 16S rDNA sequence of strain MBCN152-1 shared the greatest similarity with that of *S. humidus* (99%) (Fig. 8). A comparison of the cultural and morphological characteristics of strain MBCN152-1 with those of *S. humidus* (46) indicated that several characteristics of strain MBCN152-1 differed from those of *S. humidus*. For example, according to Shirling and Gottlieb (46), the surface of the spores of *S. humidus* is smooth, whereas that of strain MBCN152-1 was spiny (Fig. 7). Furthermore, *S. humidus* produces a yellow pigment on ISP media, whereas strain MBCN152-1 did not produce a distinctive pigment on any of the media (Table 1). These results indicate that strain MBCN152-1 is a closely related species that is distinct from *S. humidus*.

## Discussion

*Alternaria* species have been reported to cause severe yield losses in most cruciferous crops; however, there is no proven source of transferable resistance in any of the hosts (29). The disease caused by *A. brassicicola* in cabbage plug seedlings is a growing threat to cabbage cultivation in Japan (21). The methods for disease prevention and control of seed- and airborne *A. brassicicola* are based on chemical control. After the first appearance of disease symptoms, stringent fungicide (polyoxin) application is an effective method for reducing losses (54). However, the use of synthetic chemical pesticides to control plant diseases is now less acceptable in modern agriculture because many of these pesticides are harmful to non-target species in the ecosystem and hazardous to the environment (57). An alternative protection method is the use of biocontrol, wherein beneficial organisms are actively employed to suppress the populations of harmful organisms to less than economic levels (8). Actinomycetes (particularly *Streptomyces* species) are known to produce a number of biologically active secondary metabolites and fungal cell-wall degrading enzymes such as cellulases, hemicellulases, glucanases, and chitinases (37, 50, 56). In the present study, we isolated 77 actinomycetes strains from surface-disinfected cabbage and screened for their suppressive effects on spray-inoculated *A. brassicicola*. Accordingly, strain MBCN152-1 with the highest suppressive effect and no adverse effect on seedling growth was selected as the best candidate for the biocontrol of *A. brassicicola* infection of cabbage seedlings.

The results of the greenhouse experiment showed that strain MBCN152-1 achieved significant protection against spray-inoculated *A. brassicicola* on cabbage plug seedlings. Our results are consistent with those of Shimizu *et al.* (43) who reported that the application of *Streptomyces* sp. strain MBCu-56 suppressed anthracnose disease in cucumber. These results confirmed that MBCN152-1 suppresses the development of symptoms caused by air-borne *A. brassicicola* and increases the number of viable seedlings under greenhouse conditions.

Seeds infected with *A. brassicicola* on the seed coat or mycelium under the seed coat are the main routes of distribution for the pathogen (33). Seed infection reduces germination and seedling vigor, as well as pre- and post-emergence damping-off. In the present study, the incidence of disease caused by seed-borne *A. brassicicola* was significantly lower with the soil mixing treatment with strain MBCN152-1 than with the control. However, we did not test the biocontrol efficacy of seed dressing with strain MBCN152-1. Although strain MBCN152-1 is a biocontrol agent, it may be better to reduce the amount applied as much as possible. Tahvonen *et al.* (53) reported the high efficacy of seed dressing using *Streptomyces griseoviridis* on barley and spring wheat to prevent foot rot disease. Therefore, further studies to evaluate the biocontrol effects of seed dressing are needed.

Strain MBCN152-1 is considered to be an endophyte because it is re-isolated from surface-disinfected cabbage seedlings grown in soil mix containing spores of the strain (6, 32, 34, 36, 38, 51). In addition, the endophytic colonization of seedlings by strain MBCN152-1 was confirmed by SEM. Castillo *et al.* (3) reported that SEM and environmental SEM may both be used as effective tools in the identification and biological characterization of endophytic actinomycetes. Strain MBCN152-1 multiplied on the surface of the seedlings and then entered the epidermal cells, possibly through the cell wall. Several modes of penetration by endophytic actinomycetes into their host plants have been reported. Some actinomycetes penetrate through the stomata and colonize intercellular spaces, whereas other actinomycetes penetrate through the epidermal cell wall and colonize inside plant cells (38, 51). Suzuki *et al.* (49) reported that the wax covering the undulating cuticle layer on the leaf surface of rhododendron seedlings was degraded beneath the growing hyphae of *S. galbus* MBR-5 due to the production of hydrolytic enzymes such as cellulase, xylanase, and pectinase.

Endophytic actinomycetes may produce antimicrobial metabolites within their host plants because actinomycetes such as *Streptomyces* species are known for their ability to produce biologically active secondary metabolites, particularly antibiotics (28). However, Castillo *et al.* (3) isolated 39 endophytic actinomycetes and found that most of these isolates possessed no detectable antibiotic properties; however, at least seven exhibited antibacterial and antifungal activities. The antagonism of strain MBCN152-1 against *A. brassicicola* was examined on agar media. Dual culture tests demonstrated that the strain lacked the ability to produce antibiotics with antifungal activity toward *A. brassicicola*. Furthermore, the germination of *A. brassicola* conidia was not inhibited on the surface of seedlings pretreated with strain MBCN152-1. Therefore, we consider another mechanism such as induced resistance to play an important role in disease suppression. The ability of endophytic actinomycetes to induce systemic resistance in host plants has been reported in several studies. *S. galbus* strain MBR-5 induces disease resistance and the expression of the PDF1.2 gene, thereby leading to the accumulation of a phytoalexin in *Arabidopsis thaliana*, which protects it from the anthracnose disease caused by *Colletotrichum higginsianum* (42). In addition, Conn *et al.* (5) investigated the expression of salycilic acid (SA)-, jasmonate (JA)-, and ethylene (ET)-inducible genes in *A. thaliana* when inoculated with endophytic actinomycetes (*Streptomyces* sp. strains EN27 and EN28, *Micromonospora* sp. strain EN43, and *Nocardioides albus* EN46). All of these endophytes “primed” the SA and JA/ET pathways, which are linked to systemic resistance, by up-regulating pathogenesis-related proteins and other antimicrobial proteins that are effective against several pathogens.

Tian *et al.* (55) analyzed the 16S rRNA genes of 45 and 33 clones of endophytic actinomycete isolates obtained from the roots and stems, respectively, and found that the clones obtained from the roots were affiliated with nine genera of known actinobacteria. We found that the 16S rDNA sequence of strain MBCN152-1 shared the greatest similarity with that of *S. humidus*. However, the cultural characteristics of the strain differed from that of *S. humidus*. *S. humidus* produced a yellowish diffusible pigment on ISP media, whereas no pigments were produced by MBCN152-1 (46). According to SEM observations, the surface of MBCN152-1 spores was spiny, whereas that of *S. humidus* spores is smooth. Based on differences in the homologies of the 16S rDNA sequences of the nearest matching type strains, as well as morphological differences and cultural characteristics, we consider strain MBCN152-1 to be a *S. humidus*-related species, which is distinct from *S. humidus*. A phylogenetic analysis showed that strain MBCN152-1 grouped into a different cluster from a clade containing plant pathogenic *Streptomyces* species such as *S. scabies*, *S. turgidiscabies*, and *S. acidiscabies*, thereby indicating that MBCN152-1 is not a plant pathogenic species.

*A. brassicicola* causes disease in a wide range of cruciferous plants such as broccoli and white cabbage. The production of these plants using plug systems is gradually becoming more popular in Japan; thus, the incidence of *A. brassicicola* in these plants may become more serious. The results of the present study indicate the potential of strain MBCN152-1 of an endophytic *Streptomyces* to control *A. brassicicola* during seed-borne and secondary infection stages. Therefore, strain MBCN152-1 may be used as a promising biological control agent to protect against *A. brassicicola* on plug seedlings in various *Brassica* crops.

## Figures and Tables

**Table 1:** Effects of strain MBCN152-1 on the hyphal growth of *Alternaria brassicicola* on agar media

Media	Colony radius (mm)^a^	Fungal growth inhibition	

		Width of the inhibition zone (mm)^b^	Reduced rate (%)
1.5% water agar	≥45	0	0
IMA-2	≥45	0	0
Bennet	≥45	0	0
PDA	≥45	0	0

a Radius of the *A. brassicicola* colony in the dual culture plate.

b The width of the zone of inhibition between both colonies.

**Table 2:** Re-isolation frequency of strain MBCN152-1 from cotyledon leaves of cabbage seedlings

Samples^a^	Re-isolation frequency (%)^b^
Untreated control	
Unsterilized cotyledon leaves	0
Surface-disinfected cotyledon leaves	0
MBCN152-1 treatment	
Unsterilized cotyledon leaves	100
Surface-disinfected cotyledon leaves	33

a Cotyledon leaves were detached from seedlings 7 d after sowing. Half of the cotyledon leaves were surface-disinfected with ethanol and sodium hypochlorite before an incubation on 1.5% water agar.

b Re-isolation frequency (%)=No. of cotyledon leaves from which strain MBCN152-1 was isolated/Total no. of cotyledon leaves×100.

**Table 3:** Cultural characteristics of strain MBCN152-1 colonies on five different International *Streptomyces* Project (ISP) media.

Medium	Feature	Characteristics
ISP-2	Aerial mycelium	Abundant, powdery, olive gray
	Substrate hyphae	Light reddish yellow
	Reverse of the substrate mycelium	Light reddish yellow
	Diffusible pigment	None

ISP-3	Aerial mycelium	Abundant, powdery, medium gray
	Substrate hyphae	Yellowish brown
	Reverse of the substrate mycelium	Light grayish brown
	Diffusible pigment	None

ISP-4	Aerial mycelium	Abundant, powdery, medium gray
	Substrate hyphae	Light reddish yellow
	Reverse of the substrate mycelium	Light reddish yellow
	Diffusible pigment	None

ISP-5	Aerial mycelium	Abundant, powdery, purplish gray
	Substrate hyphae	Pale reddish yellow
	Reverse of the substrate mycelium	Pale reddish yellow
	Diffusible pigment	None
